# The difference between semi-continuum model and Richards’ equation for unsaturated porous media flow

**DOI:** 10.1038/s41598-022-11437-9

**Published:** 2022-05-10

**Authors:** Rostislav Vodák, Tomáš Fürst, Miloslav Šír, Jakub Kmec

**Affiliations:** 1 Department of Mathematical Analysis and Applications of Mathematics, Faculty of Science, Palacký University in Olomouc, Olomouc, 779 00 Czech Republic; 2 Joint Laboratory of Optics, Faculty of Science, Palacký University in Olomouc, Olomouc, 772 07 Czech Republic

**Keywords:** Hydrology, Mathematics and computing

## Abstract

Semi-continuum modelling of unsaturated porous media flow is based on representing the porous medium as a grid of non-infinitesimal blocks that retain the character of a porous medium. This approach is similar to the hybrid/multiscale modelling. Semi-continuum model is able to physically correctly describe diffusion-like flow, finger-like flow, and the transition between them. This article presents the limit of the semi-continuum model as the block size goes to zero. In the limiting process, the retention curve of each block scales with the block size and in the limit becomes a hysteresis operator of the Prandtl-type used in elasto-plasticity models. Mathematical analysis showed that the limit of the semi-continuum model is a hyperbolic-parabolic partial differential equation with a hysteresis operator of Prandl’s type. This limit differs from the standard Richards’ equation, which is a parabolic equation and is not able to describe finger-like flow.

## Introduction

A porous medium is a solid material which contains pores. Pores are filled with one or more different fluids—liquids and gases. The skeletal structure of a porous medium (called the matrix) has usually a very complex geometry. In soil physics, the porous medium is called *saturated* if all the pores contain a liquid (usually water), and *unsaturated* if some pores are filled with liquid and some with gas (usually air). Saturation is defined as the ratio of the liquid volume and the pore volume. There are many important applications of porous media flow modelling, such as oil recovery^1^ and soil physics^2,3^. The infiltration of rainwater into the soil and the recharge of groundwater reserved by soil water form the basis of the hydrological cycle of land. In this paper, we present a limit of the semi-continuum model^4^ that describes the infiltration of water into a porous material such as soil.

In contrast to “bulk” fluid flow (described, e.g., by the Navier–Stokes equations), inertial forces in the porous medium can usually be neglected due to small flow velocities. However, the porous media flow may still be very complicated because the liquid movement in the porous matrix depends on the pressure in the wetting fluid, which is determined by the capillary forces^5,6^ which, in turn, are directly affected by the geometry of the porous matrix^7^.

In an unsaturated porous medium, capillary pressure and saturation are related by a material characteristics—the so-called *retention curve*—which is known to exhibit substantial hysteresis^8^. The retention curve gives the matrix potential (or capillary pressure) as a function of saturation under equilibrium conditions^9,10^. It was shown that the retention curve and other physical properties depend on the volume of the sample^11–15^. However, this important issue has not usually been considered in porous media flow modelling.

Three different approaches to porous medium flow modelling can be identified: (1) Continuum modelling, which is based on the idea of representing the porous medium as a continuum using the concept of the representative elementary volume (REV)^16^. REV is the smallest volume for which the saturation and pressure fields (and other characteristics) can still be considered smooth, so that these characteristics provide a representative value of the whole. A representative value cannot be defined for smaller volumes than REV. In this concept, the key physical quantities (saturation and capillary pressure) are considered scalar fields smoothly varying in (continuous) time and space. (2) Percolation theory, in which the pore space is described as a network of nodes and bonds^17^. All physical quantities (saturation, pressure, and time) are usually considered discrete in these models: The saturation is either one (full pore) or zero (empty pore), and the course of infiltration is followed in discrete time steps—n each step a single pore is filled or emptied. (3) Semi-continuum modelling, in which the porous medium is represented as a grid of non-infinitesimal blocks that retain the character of a porous medium^4,18^. Saturation and capillary pressure are assumed continuous in time but constant within each block (i.e. piecewise constant in space), the time is either continuous or discrete.

In soil physics, three different descriptions of liquid transport through the porous material have been used. (A) At the continuum and semi-continuum level, the movement of a liquid through the porous body has usually been described by the Darcy-Buckingham law^19^. (B) At the level of capillary pores, various displacement rules have been proposed^20–22^. (C) At the level of large, non-capillary pores, the kinematic wave equation has been used^23^.

As a result, three different modelling approaches (1–3) may be combined with at least three flow rules (A–C). Thus, a plethora of flow models are used to describe fluid flow in various types of porous media^17,24–30^. The oldest model is a combination (1A), which is expressed by the Richards’ Equation (RE)^31^. It has long been known that this model works well only for diffusion-like flow and fails to describe finger-like flow^32,33^. Therefore, especially in the field of soil physics and soil hydrology, many attempts were made to modify the RE so that it may also capture finger-like flow^34–44^. Another attempt is the combination (3A) proposed in^4,18,45,46^. It has been shown that some of these models describe diffusion-like flow, finger-like flow, and the transition between the two regimes^4,46^.

Models (1A) and (3A) are very similar—they both use a continuous method of modelling the discrete porous material and they both use an almost identical form of the Darcy–Buckingham law to describe liquid transfer. Therefore, the question arises: How do they differ? This article seeks answers to this question. To make the argument as clear as possible, we present the one-dimensional form of the problem. The main characteristic of finger flow is the saturation overshoot^47,48^. Thus in 1D, we concentrate on the ability of the model to capture the saturation overshoot.

In this article, (1) we consider the limit of the semi-continuum model^4^ as the block size goes to zero. Because the retention curve depends on the physical dimension of the block, (2) we introduce a physically relevant scaling of the retention curve so that the slope of the retention curve decreases with the sample size. (3) We introduce a novel type of porous media flow equation which is obtained as the limit of the semi-continuum model using appropriate scaling of the retention curve. (4) We analyse how this equation differs from the RE.

## Methods

### Continuum modelling of porous media flow: Richards’ equation

The Darcy–Buckingham law is the key constitutive relationship for modelling flow and transport in the saturated and unsaturated porous medium^16^. The flux *q* (m/s) modeled by the Darcy–Buckingham law takes the form:

1 $$\begin{aligned} q = \frac{\kappa }{\mu } k(S) (\rho g - \nabla P ), \end{aligned}$$

where $\kappa$ (m^2^) denotes the intrinsic permeability, $\rho$ (kg/m^3^) the fluid density, *g* (m/s^2^) acceleration due to gravity, μ (Pas) the dynamic viscosity of fluid, and *P* (Pa) the capillary pressure in the unsaturated medium (caused by capillarity) and the hydrostatic pressure in the saturated medium (caused by gravity). Capillary pressure in a liquid is created by the action of capillary menisci. The capillary meniscus is the curved surface forming the interface between the liquid phase (water) and gas (air) caused by surface tension. Pressure in the liquid phase is determined by the curvature of the menisci by means of the Young–Laplace equation^49^—tension in the liquid phase increases with the curvature of the menisci. The contact angle at the meeting points of all three phases (liquid, gas, and solid matrix) forms the boundary condition for the Young–Laplace equation.

There are two material characteristics in the unsaturated medium, the retention curve and the function *k*(*S*) (–), which is called the relative permeability. Both these material characteristics have to be measured. Many different models of the retention curve and the relative permeability function exist^16,50^.

RE is then obtained by the combination of the Darcy–Buckingham law and the mass balance law equations^16^ and is usually stated in the following form in 1D:

2 $$\begin{aligned} \theta \partial _t S = \partial _x \Big ( \frac{\kappa }{\mu } k(S) \big ( \partial _x P(S) - \rho g \big ) \Big ), \end{aligned}$$

where $\theta$ (–) denotes the porosity of the material. We use the notation

$$\begin{aligned} \partial _t : = \frac{\partial }{\partial t} \qquad \text{ and } \qquad \partial _x : = \frac{\partial }{\partial x}. \end{aligned}$$

From the mathematical point of view, RE is an elliptic partial differential equation of the second order for a saturated porous medium (*P* is the hydrostatic pressure in this case) and is a parabolic partial differential equation of the second order for an unsaturated porous medium^16^. In summary, RE consists of the law of mass conservation, together with a constitutive relationship for the liquid flow, and two material characteristics.

### Semi-continuum modelling of porous media flow

The semi-continuum modelling of fluid transfer in porous media is based on representing the porous medium as a grid of non-infinitesimal blocks that retain the character of a porous medium. Many soil science researchers have tested this idea. As soon as 1989, Glass and Yarrington^18^ proposed a cellular automaton under the title of “mechanistic modelling”, or “Macro Modified Invasion Percolation”.

In Kmec et al.^4,46^, the authors introduced a semi-continuum model which will be described in this section. Let us consider a long narrow vertical test tube of cross-section *A* (m^2^) and height *L* (m) filled with homogeneous and isotropic porous medium. We divide the tube into blocks of height $\Delta x$. These blocks represent “pieces” of the original porous medium in the sense that each block is characterized by its retention curve, porosity, and permeability. The tube now consists of slices [$i \Delta x, (i + 1)\Delta x$] with $i = 0, 1, \ldots ,N$. The key quantities that we want to track are:

- Saturation *S* (–) in each block.
- Capillary pressure *P* (Pa) in each block.
- Flux $q_{i,i+1}$ (m/s) between the blocks *i* and $i+1$. Volumetric fluxes in [$\hbox {m}^{3}/\hbox {s}$] can be recovered as $A q_{i,i+1}$. Naturally, only fluxes between pairs of neighboring blocks are nonzero in this setting.

At each instant, the pressure $P=P(x,t)$ and saturation $S=S(x,t)$ are considered constant within each block. We thus use the notations $S_i(t)=S(x,t)$ and $P_i(t)=P(x,t)$, $x\in [i \Delta x,(i+1)\Delta x)$. Gravity is directed downward along the long axis of the tube, which is called the x-axis here. A constant (in time) influx $q_0$ (m/s) is set across the top boundary (at $x = 0$). Zero discharge $q_L = 0$ (i.e. zero flux) is assumed at the bottom boundary (at $x = L$). Saturation and pressure in each block, and the fluxes across the block boundaries are considered continuous in time, however, to solve the model numerically, time is discretized with a step $\Delta t$. At each time step, the saturation in each of the blocks is updated according to Eq. (4), which is an explicit discretized version of the semi-continuum model [Eq. (3)]

3 $$\begin{aligned} \theta \partial _t S_i(t) =\frac{1}{\Delta x} \left[ q_{i-1,i}(t) -q_{i,i+1}(t) \right] , \end{aligned}$$

4 $$\begin{aligned} \frac{\theta }{\Delta t} \left[ S_i(t) - S_i(t-\Delta t) \right] = \frac{1}{\Delta x} \left[ q_{i-1,i}(t-\Delta t) - q_{i,i+1}(t-\Delta t) \right] . \end{aligned}$$

A backward time discretization of Eq. (3) can be also used (see the Discussion in^46^).

**Figure 1:**
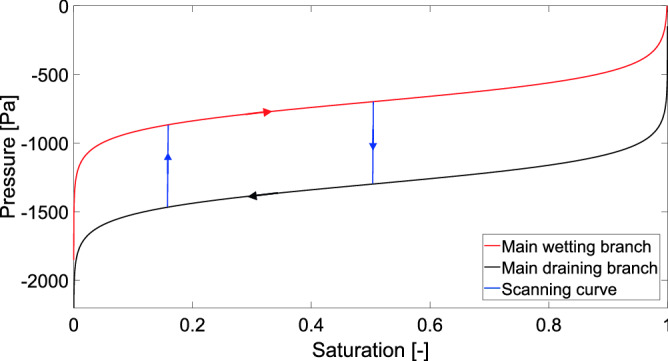
Model of the retention curve hysteresis. The coloured lines correspond to different parts of the retention curve.

Next, the capillary pressure in each block is updated according to the retention curve. There are many approaches to modelling hysteresis^51–53^, but here the hysteresis is modelled by the simplest approach possible: If a block switches from draining to wetting, the capillary pressure starts moving from the draining branch toward the main wetting branch of the retention curve along a straight line with a very large gradient $K_{PS}$^8,54,55^. Once the block (now in wetting mode) reaches the main wetting branch, it sticks to it and continues along it (see Fig. 1). This approach to hysteresis is motivated by the Prandtl-type hysteresis operator^56^ and is similar to the play-type hysteresis used e.g. in^57^. Modelling the scanning curves as lines with large slopes is a numerically feasible realization of the idea that the pressure in the block “jumps” from the draining branch to the wetting branch without any accompanying change in saturation. All it takes to produce a large increase in the liquid pressure (i.e., a decrease in matrix suction) is to relax (i.e., increase the radius of) the capillary menisci supporting the water body. This may be achieved with a negligible amount of liquid, thus keeping the saturation almost unchanged. This mechanism explains the hysteresis in the retention curve and offers the above-described modelling strategy.

The last step of the modelling loop is the update of the fluxes. In the fingering regime, it is crucial how the model treats the conductance at the finger tip, where $\nabla P$ is large, and *S* changes abruptly from small values in front of the fingertip to large values inside the finger. The semi-continuum model uses the following discretization of the Darcy–Buckingham law:

5 $$\begin{aligned} q_{i,i+1}(t) = \frac{\kappa }{\mu } \sqrt{k(S_i(t)) k(S_{i+1}(t))} \left( \rho g - \frac{P_{i+1}(t) - P_i(t)}{\Delta x} \right) . \end{aligned}$$

Thus, for the relative permeability across the boundary of two adjacent blocks *i* and $i+1$, we simply take the geometric mean of the permeability of the respective blocks. The geometric mean $\sqrt{ab}$ has the desirable property of being small if one of the numbers *a* or *b* is small. It is also possible to use the harmonic mean with similar results. There is a theoretical justification for using this type of averaging^58^.

The semi-continuum model^4^ works as follows:

1. The size of the blocks $\Delta x$ is chosen and an appropriate time step $\Delta t$ is set. Initial saturation is prescribed in each block, the corresponding capillary pressure is computed from the retention curve, and all fluxes are initially set to zero.
2. The top boundary condition is set: The flux into the topmost block is set and fixed to $q_0$. The bottom boundary condition is set: The flux out of the bottom block is set and fixed to zero.
3. Using the current value of the fluxes $q_{i-1,i}$ and $q_{i,i+1}$, saturation $S_i$ in each block is updated according to Eq. (4).
4. Pressure $P_i$ in each block is updated according to retention curve and hysteresis model, keeping track whether the block is in the imbibition or draining mode.
5. Fluxes $q_{i,i+1}$ between neighboring blocks are updated according to Eq. (5), keeping the boundary fluxes fixed by step 2.
6. Time is updated to $t + \Delta t$ and the process goes back to step 3.

The fundamental difference between a numerical scheme for the RE and the semi-continuum model lies in the scaling of the retention curve. In a numerical scheme for RE, the retention curve remains the same as the block size goes to zero. In the semi-continuum model, the slope of the retention curve vanished with vanishing block size. Thus, with $\Delta x \rightarrow 0$, the semi-continuum model does not converge to the RE.

## Results

In Kmec et al.^4^, the authors showed that the model is able to reproduce well all the observed features in unsaturated flow in a narrow vertical test tube filled with sand in experiments reported by DiCarlo^47,48,59^. The model correctly predicts when a saturation overshoot effect will appear. Moreover, it captures well both the interesting aspects of the overshoot behavior: (1) the non-monotonic dependence of the overshoot magnitude on the influx, and (2) the transition from the overshoot regime to diffusion-like regime for increasing initial saturation. See^4^ for all the details.

Fingering regime in a narrow tube does not allow the fingers to exhibit complicated spatial patterns. To observe these, one has to switch to two-dimensional experiments such as^18,32,60–65^. It is straightforward to extend the semi-continuum model to two spatial dimensions. However, to keep the setting as clear as possible, we stick to the simplest 1D formulation in this text. In^46^, the authors used the two-dimensional version of the model to show that it is able to correctly reproduce the transition between a fingering regime with saturation overshoot (for small initial saturation) and a diffusion-like regime of a stable flat water front with a monotonic saturation profile (for large initial saturation)—see Fig. 6 in^46^.

We can clearly see that the simulations of the semi-continuum model are in good agreement with the experimental results. However, the crucial question how to choose the block size is still missing. This is clearly a “parameter” of the semi-continuum model, and clearly a rather artificial one.

**Figure 2:**
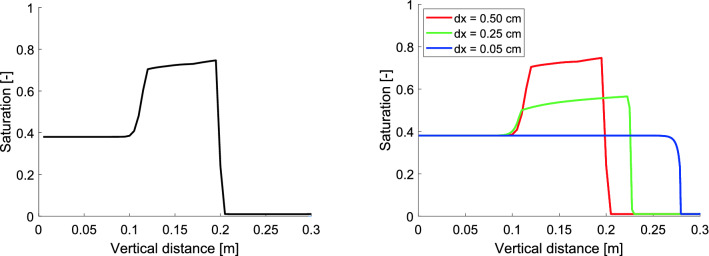
Left panel: The dependence of the moisture profile at $t = 10$ mins on time step $\Delta t$, the profiles are almost identical for a range of $\Delta t$ between $10^{-3}$ and $10^{-5}$. Right panel: The dependence of the moisture profile at $t = 10$ minutes on $\Delta x$. As $\Delta x \rightarrow 0$, without proper scaling of the retention curve, the overshoot behavior would disappear. The parameters used for the simulations are given in Table 1.

First, note that the time step is not a free parameter of the model—it is a discretization parameter. Figure 2 (left) shows the behavior of the 1D semi-continuum model for a range of $\Delta t$ values. The solution is stable in the limit $\Delta t \rightarrow 0$. This is not the case for $\Delta x$. If we let the block size go to zero ($\Delta t$ has to go to zero, too), and kept the retention curve constant, the overshoot behavior would disappear (see Fig. 2 right).

In the limiting process mentioned above (Fig. 2), the dependence of the retention curve on the block size^11,13^ is missing. We argue that this is the key ingredient which is usually missing in the RE-based models and their modifications. Therefore, in the semi-continuum model, the dependence of the retention curve on the block size $\Delta x$ is implemented. This procedure is named *scaling of the retention curve* and will be discussed in the next section.

### Scaling of the retention curve

The scaling of the retention curve must respect a physically justified requirement that the nature of the flow does not change when the block size is changed. This means that the fluxes across the block boundaries must stay roughly the same when $\Delta x$ changes. The fluxes are given by Eq. (5) in which decreasing $\Delta x$ by half increases the flux by a factor of two. To compensate for this, the difference in pressure between the blocks must decrease. Decreasing the pressure difference without changing the saturation difference amounts to the flattening of the retention curve. Based on this idea, we propose the following simple scaling mechanism in which the main branches of the retention curve of a block take the form

6 $$\begin{aligned} P_w(h,S) = h \Bigg (-100 \, \log \Big (\frac{1}{S}-1\Big )\Bigg ) + C_1, \end{aligned}$$

for the main wetting branch, and

7 $$\begin{aligned} P_d(h,S) = h \Bigg (-100 \, \log \Big (\frac{1}{S}-1\Big )\Bigg ) + C_2, \end{aligned}$$

for the main draining branch, where $C_1$ (Pa) and $C_2$ (Pa) are constants. The parameter *h* (m) is the scaling parameter equals to the block size $\Delta x$. Notice that in the scaling process, the distance between the two main branches does not change (although this feature is not a crucial requirement for further consideration). More standard models of the retention curve may also be used in the scaling process, e.g., the van Genuchten equation^50^.

**Figure 3:**
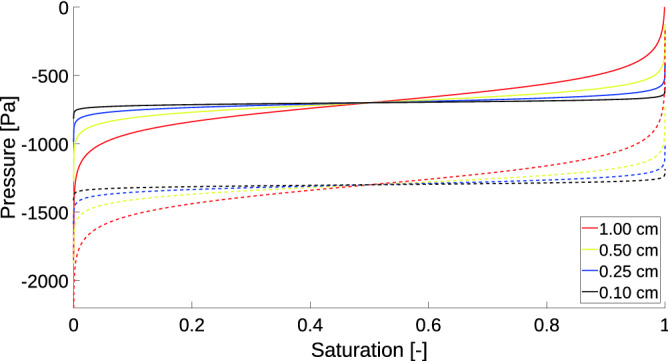
Scaling of the retention curve with the block size $\Delta x$. Grid block sizes are colour-coded. The solid line denotes the main wetting branch and the dashed line denotes the main draining branch.

Let us explain, by means of an example of a porous material with a simple pore structure, how the shape of the retention curve changes during scaling. The retention curve given by Eqs. (6) and (7) with $C_1=-700$ Pa and $C_2=-1300$ Pa roughly matches the main branches of retention curve of 20/30 sand in the experiments of DiCarlo^47^. Figure 3 illustrates the scaling of this retention curve as $\Delta x$ goes to zero. As $\Delta x$ decreases, the slope of both branches of the retention curve decreases, too. In the limit, both branches take the form of horizontal lines.

In practice, the retention curve will be measured for a sample of a known dimension $h_{ref}$. The scaling is then given by Eqs. (6) and (7) and there is no need to measure the retention curve repeatedly for blocks of different dimensions.

### Convergence of the moisture profiles

**Figure 4:**
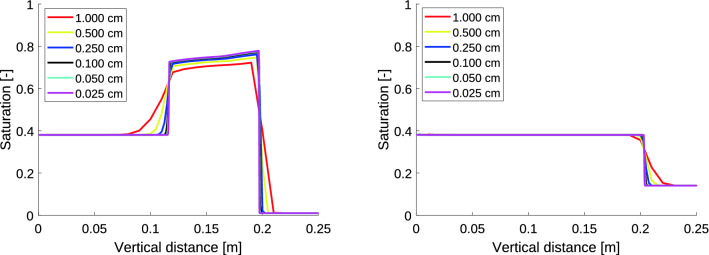
Left panel: Convergence of the moisture profile at $t = 10$ minutes for $\Delta x \rightarrow 0$ for initial saturation $S_{i, in}=0.01$, $i=0,\ldots ,N$ and constant top boundary flux $q_0 = 6 \times 10^{-5}$ m/s. The flux out of the bottom block is set to zero. Each colour corresponds to a particular grid block size. The moisture profile converges and retains the overshoot pattern. Right panel: Convergence of the moisture profile at $t = 5$ minutes for $\Delta x \rightarrow 0$ for initial saturation $S_{i, in}=0.14$, $i=0,\ldots ,N$ and constant top boundary flux $q_0 = 6 \times 10^{-5}$ m/s. The flux out of the bottom block is set to zero. Each colour corresponds to a particular grid block size. The moisture profile converges to a sharp water-front without saturation overshoot.

**Figure 5:**
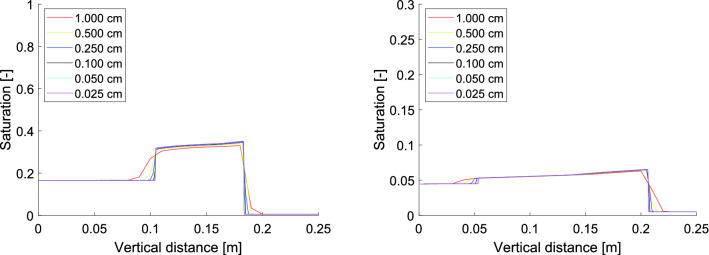
Left panel: Convergence of the moisture profile at $t = 50$ minutes for $\Delta x \rightarrow 0$ for initial saturation $S_{i, in}=0.01$, $i=0,\ldots ,N$ and constant top boundary flux $q_0 = 5 \times 10^{-6}$ m/s. The flux out of the bottom block is set to zero. Each colour corresponds to a particular grid block size. The moisture profile converges and retains the overshoot pattern. Right panel: Convergence of the moisture profile at $t = 600$ minutes for $\Delta x \rightarrow 0$ for initial saturation $S_{i, in}=0.14$, $i=0,\ldots ,N$ and constant top boundary flux $q_0 = 1 \times 10^{-7}$ m/s. The flux out of the bottom block is set to zero. Each colour corresponds to a particular grid block size. A smaller *y*-axis scale is used to show the details of the moisture profiles. The moisture profile converges and retains the overshoot pattern.

Let us show that the proposed scaling of the retention curve indeed does not change the nature of the flow. A simulation of liquid infiltration into a vertical column of porous material with a constant top boundary influx $q_0$ is shown in Fig. 4. The parameters used for the simulations are given in Table 1. Since we are not interested in the bottom part of the porous medium, this part is not depicted here. Left panel of Fig. 4 shows the calculated moisture profiles for the initial saturation $S_{i, in}=0.01$ for a decreasing sequence of block size values. Right panel of Fig. 4 shows the numerical convergence for $\Delta x \rightarrow 0$ in case of a diffusion-like flow regime (the initial saturation $S_{i, in}=0.14$). Moreover, the moisture profile convergence is not affected by the different top boundary flux. Figure 5 shows numerical convergence for $\Delta x \rightarrow 0$ in the case of two different top boundary fluxes varying in two orders of magnitude. A different scale of the *y*-axis is used in the right panel of Fig. 5 to show the details.

We may observe that this retention curve scaling preserves the character of the flow across all levels of $\Delta x$ both in the finger regime and the diffusion-like regime. Therefore, the semi-continuum model allows for a physically reasonable scaling of the retention curve. Note that such considerations are impossible when deriving the RE.

**Table 1:** Parameters used for the simulations presented in Fig. 4.

Parameter	Symbol	Value
Porosity	$\theta$	0.35
Density of water	$\rho$	1000 kg/m^3^
Dynamic viscosity of water	$\mu$	$9 \times 10^{-4}\, \hbox {Pas}$
Intrinsic permeability	$\kappa$	$1 \times 10^{-10}\, \hbox {m}^{2}$
Relative permeability	*k*(*S*)	$S^3$
Acceleration due to gravity	*g*	$9.81 \,\hbox {m}/\hbox {s}^{2}$
Large gradient	$K_{PS}$	$10^5$

**Figure 6:**
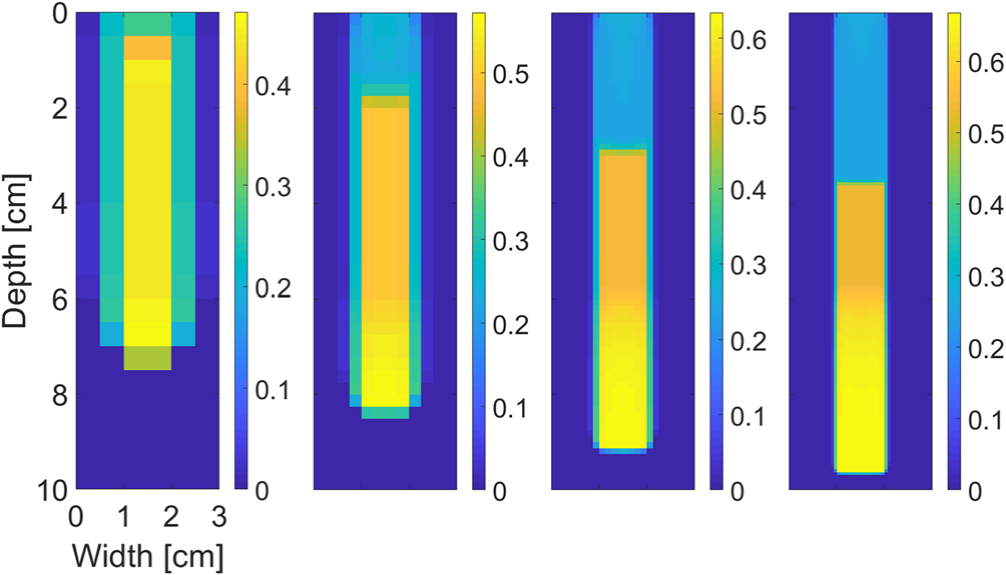
Convergence of the moisture profile in 2D at time $t = 14.5$ minutes for $\Delta x = 0.500$ cm, 0.250 cm, 0.125 cm and 0.0625 cm from the left to the right for initial saturation $S_{in}=0.002$. The moisture profile converges and retains the overshoot pattern. Saturation values are colour-coded according to the colour bar on the right.

The scaling of the retention curve described above can be also demonstrated in the 2D version of the semi-continuum model presented in^46^. Figure 6 shows the predicted moisture profile for 2D simulation for a decreasing sequence of block size values. Notice analogous behaviour to the 1D case: The saturation in the fingertip increases slightly with decreasing block size, but the oversaturated zone remains roughly the same. However, due to numerical errors that are not present in 1D, the fingers become more narrow and consequently slightly faster. The limiting process in 2D requires more attention and will be addressed in a subsequent paper. The numerical evidence of the 1D and 2D cases suggests there should be a limit form of the semi-continuum model, i.e., a model should exist to which the semi-continuum model converges as $\Delta x \rightarrow 0$, if the retention curve is scaled in this appropriate way.

### Limit of the semi-continuum model

With this scaling of the retention curve, we can finally introduce the limit of the semi-continuum model. The limit of the model derived here is a formal one, i.e. we *assume* the solution of the semi-continuum model converges as $\Delta x \rightarrow 0$ to a function and show which equation this function should satisfy. This equation is called the *limit of the semi-continuum model*. A detailed derivation of this limit is given in the Supplementary Information.

8 $$\begin{aligned}&\left( K_{PS}\partial _t S - \partial _t P_H \right) \left( P_H - v \right) \ge 0, \qquad \text{ for } \text{ all } v \in [C_2, C_1], \qquad \text{ and } P_H \in [C_2, C_1], \end{aligned}$$

9 $$\begin{aligned}&\theta \partial _t S +\partial _x\left( \frac{\kappa }{\mu } \sqrt{k(S^-)}\sqrt{k(S^+)} (\rho g - \partial _x P_H)\right) = 0, \qquad S^{\pm }(x_0,t) =\lim _{x\rightarrow x_0 ^\pm }S(x,t). \end{aligned}$$

Equations (8) and (9) represent the classical form of the limit of the semi-continuum model. It is a partial differential equation containing a Prandtl-type hysteresis operator $P_H$ [defined by Eq. (8)] under the derivative. If we are located on the main wetting or draining branches, the limit will be a hyperbolic differential equation. In this case, the pressure-saturation relation is constant and thus independent on the space variable. This makes the limit switch between parabolic and hyperbolic type. Let us also note that if the saturation is continuous, then $k(S) = \sqrt{k(S^-)}\sqrt{k(S^+)}$.

Similar notation as in equation (9) is also used for the classical RE [see Eq. (10)].

10 $$\begin{aligned} \theta \partial _t S +\partial _x\left( \frac{\kappa }{\mu } k(S) (\rho g - \partial _x P(S))\right) = 0. \end{aligned}$$

The two equations differ in how the relationship between pressure and saturation is expressed. In the *limit of the semi-continuum model* [Eq. (9)], the pressure-saturation relation is defined by a Prandtl-type hysteresis operator $P_H$, while for the RE [Eq. (10)], the pressure-saturation relation is described by a hysteretic operator *P* with wetting and draining branches represented by monotonically increasing functions. Moreover, Eq. (9) preserves the geometric mean of the relative permeability.

In light of the analysis above, we argue that the RE arises from a physically unsound limit of the semi-continuum model which ignores the proper scaling of the retention curve. Thus, it is not surprising that the RE is unable to capture overshoot behavior. If the proper scaling of the retention curve is included, we obtain a different limit and the overshoot behavior is not lost.

## Discussion

The limit of the semi-continuum model [Eqs. (3), (4), (5)] was found by means of mathematical analysis (see Supplementary Information) in the form of a partial differential Eq. (9). However, the limiting process is inspired by a numerical consideration that the flow between adjacent blocks should remain roughly the same when the block size decreases. From the Darcy–Buckingham Eq. (5) it follows that the shape of the retention curve must be scaled according to the block size [Eqs. (6), (7)].

The process of *scaling the retention curve* is not a common practice in flow modelling. Therefore, its physical justification should be addressed. The question is whether the shape of the retention curve depends on the volume of the sample. This issue has been examined in the literature for a long time. The discrepancy between retention curve models and the actual measurements was already mentioned more than 60 years ago by Fatt^66^. The effects of sample dimension on capillary pressure have since then been pointed out e.g. in^11–13,67–69^. In our view, the explanation is quite simple: The main draining branch is usually measured in a pressure plate extractor. In the case of the pressure plate test^70,71^, water is extruded by gas. During the test, gas first invades large pores and displaces water there. With increasing pressure, the gas enters smaller and smaller pores gradually^70,71^. However, the topology of the porous medium is usually ignored although it plays a crucial role. During drainage, certain pores are “candidates for draining” because the applied air pressure is greater than the capillary pressure holding the water inside the pore. However, some of these pores may not be accessible because they are not connected to the body of advancing air, or water is not able to leave them because they are not connected to the body of retreating water^13^. Pražák et al.^70^ used percolation theory to model a simple capillary network. He showed that the retention curve is a constant for a homogeneous network (i.e., a network with zero variability in pore size). There is an analogy between the proposed scaling of the retention curve and such a homogeneous network. By decreasing the sample size, the variability of the pore sizes inside the sample also decreases (i.e, is more homogeneous) and thus the retention curve becomes flatter. This was experimentally confirmed in^14^.

**Figure 7:**
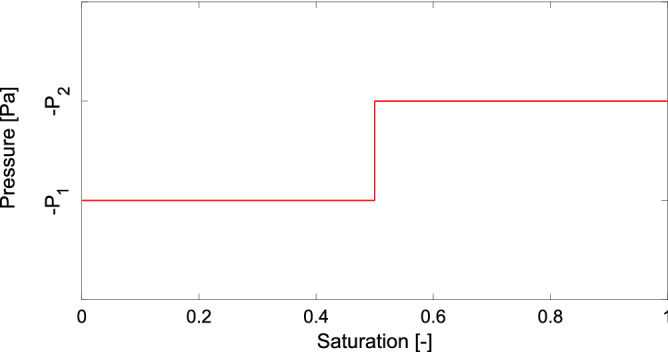
The main draining branch of two pores of radii $R_1 < R_2$ and the corresponding pressures $-P_1$ and $-P_2$. Both pores are assumed to have the same volume, thus the horizontal line is broken at $S=0.5$.

Let us perform the following thought experiment: Imagine a single pore consisting of a cylinder of radius *R*. Assuming zero contact angle, a drop of liquid “sit” inside this capillary cylinder bounded by two hemispherical menisci of radius *R*. According to the Young–Laplace equation^49^, the pressure drop across both menisci is $2 \sigma /R,$ where $\sigma$ is the surface tension of the liquid. Setting the gas pressure to zero, the liquid drop is under tension (i.e., negative pressure) $P = 2 \sigma /R$. Connecting such an empty pore to a liquid reservoir at a pressure lower than $-P$ will yield zero saturation in the pore—the suction of the pore is not enough to draw any liquid inside. Once the pressure in the reservoir increases above $-P$, the pore will immediately fill with the liquid switching from zero to unit saturation. Thus, the dependence of saturation on pressure (i.e., the retention curve) is a horizontal line at $-P$. Let us continue this thought experiment and consider two pores of radii $R_1 < R_2$. At a certain pressure $-P_1$, the first pore will fill in completely, and at a higher pressure $-P_2$, the second one will fill in. Thus, the retention curve of this pair of two pores becomes a broken horizontal line (see Fig. 7). A macroscopic sample of a porous medium contains many pores of various shapes. The resulting retention curve of the sample arises by assembling many horizontal lines at different levels of pressure. The main point of this excursion is to explain that as the sample size converges to zero, its retention curve has to converge to the retention curve of a single pore—i.e., to a horizontal line.

It follows from the above considerations that the retention curve depends on the size of the sample. Therefore, the scaling we propose is supported by sound physical arguments. In continuum physics, material characteristics, such as the retention curve, are related to the volume of REV. Thus, if blocks smaller than REV are used, the retention curve must be scaled. This causes substantial problems in the mathematical setting. The limit of the semi-continuum model switches between a hyperbolic equation (at points located on the main branches) and a parabolic equation (at points located on the scanning curves). It is the hysteresis operator in the limiting equation that enables to switch between two equations of different types. The situation is moderately similar to modelling the compressible fluid flow in subsonic and transsonic regions, which changes from hyperbolic to elliptic equation^72^. However, in the case of the semi-continuum limit, the hysteresis operator appears inside the Laplace operator. Such equations seem to be unexplored in current mathematics^73–75^.

The idea of taking REV into account in finite element discretization is completely unusual (at least in the mathematical community), surprisingly, it has long been used in porous media^76–79^. For instance, in^77^, the authors proposed a method for the calculation of the effective permeability of sandstone with the lattice Boltzmann finite element method. The method consists of two models—pore-scale and macro-scale. First, the permeability tensor is calculated by the lattice Boltzmann method (pore-scale model). This permeability is then used for solving the diffusion equation (macro-scale model) on a finite element mesh. A crucial step in the model is determining an appropriate finite element discretization. The authors argue that a highly refined mesh cannot be used, because the size of the elements will then be so small that the continuum approximation is no longer appropriate. Thus, the size of the REV is estimated and used as a lower limit for the size of the finite elements. This means that the computational mesh takes into account the dependence of physical parameters on the size of mesh elements.

It has long been known that macro-scale models (i.e., models larger than REV) often fail to describe experimentally observed phenomena^80,81^. This usually happens because some physical properties (e.g. concentration) vary significantly within a REV. This may be an essential information for simulations^82^. Obviously, this cannot be captured by continuum-scale equations, which cease to be sufficiently accurate. A pore-scale simulations such as lattice Boltzmann methods are used in this case, however, there are two major drawbacks that make the model impractical as a predictive tool. (1) The simulations are computationally demanding and (2) lack detailed information about the pore geometry in porous media. Therefore, a logical solution is to use a pore-scale approach where the detailed structural information of the porous medium is necessary, and to use macro-scale approaches where such detailed information are not needed. This approach to modelling is represented, e.g., by multiscale^83–86^ and hybrid algorithms^80–82,87,88^. Multiscale algorithms use pore-scale models to evaluate physical properties such as permeability, density, and/or velocity. These are then upscaled into a macro-scale model. The model presented in the previous paragraph^77^ is an example of a multiscale algorithm. On the other hand, hybrid algorithms use a slightly different approach: In regions where continuum methods fail, they are replaced by the pore-scale methods. Thus, the porous medium is divided into two disjoint domains, in which different algorithms are used. Sometimes it is even needed to use several different methods across several scales^86^. For both multiscale and hybrid algorithms, the methods are strongly dependent on the size of the porous medium. There are similarities between our semi-continuum approach and hybrid/multiscale modelling in porous media. We use a retention curve that scales with the block size; thus we use a different formula dependent on the size of the porous medium. It is possible to implement an adaptive block size; in the region where the finger-like flow occurs, small blocks can be used, and in the region where the diffusion occurs, larger blocks can be used. This is a formal analogy to hybrid modelling.

At the pore scale, the fluid motion is characterized by capillary displacement and the Darcy–Buckingham law is not longer appropriate^16,20^. The transition between two different models of water displacement is captured by the semi-continuum approach. In the limit, the main wetting and draining branches degenerate into parallel lines which results in the Darcy’s law taking the character of capillary displacement. Indeed, water fills and drains the pores almost instantly, similarly as in percolation theory. Therefore, we actually use “different” physics for Darcy and sub-Darcy scales. This is achieved by varying the slope of the retention curve, not by explicit changes of the governing equations.

The use of the *geometric mean of conductivity* can be justified by the following consideration. When solving the fluid flow between adjacent blocks of porous material by the Darcy-Buckingham law [Eq. (1)], it is necessary to determine the conductivity between blocks at different saturation. The first idea could be to use the most common arithmetic mean. However, the arithmetic mean is applicable only for the extreme case of parallel pores^13^. In case of other extreme—pores in a series—Hunt et al.^13^ demonstrated that the harmonic mean should be used. In numerical experiments with random pore networks, Jang et al.^58^ concluded that the geometric mean of both values is the most appropriate.

A comparison of the RE (10) and the limit of the semi-continuum model (9) shows that there are two major differences between them. In the semi-continuum model (1) a proper scaling of the retention curve is used, and (2) the geometric mean of the conductivity between adjacent blocks is maintained. Note that the hysteresis has to be included in the retention curve because it is known^64^ that the saturation overshoot is a consequence of the retention curve hysteresis. With (1) and (2), any reasonable numerical solution of the RE will converge to the limit of the semi-continuum model. In this case, it will correctly simulate the diffusion-like flow, the finger-like flow, and the transitions between them, just as the semi-continuum model can.

## Conclusion

A semi-continuum model for the description of unsaturated homogeneous porous media flow is presented. One can see the similarity between the proposed model and the multiscale/hybrid modelling. The model is based on the idea of Macro Modified Invasion Percolation, in which the porous material is divided into blocks of non-infinitesimal size. Each block is represented by its retention curve and relative permeability. Saturation and pressure are considered continuous but constant within each block. Flow between adjacent blocks is described by the Darcy–Buckingham law. The limit of such a semi-continuum model is similar to the standard Richards’ equation. However, it differs in the way the pressure saturation relationship is captured. The retention curve has to be scaled appropriately to the size of the block. This results in a Prandtl-type hysteresis operator appearing under the derivative in the limiting equation. Moreover, the geometric mean of adjacent blocks is maintained. This limit differs from the standard RE, which is not able to describe finger-like flow. However, the physics behind both RE and the semi-continuum model is almost the same. Thus, the limit introduced above can be viewed as a reformulation of the RE in such a way that it does not lose the ability to describe finger-like flow. We conclude that the RE should be reconsidered by means of appropriate modelling of the hysteresis and proper scaling of the retention curve.

The limit of the semi-continuum model defined by Eqs. (8) and (9) represents a rather interesting mathematical object. From a mathematical point of view, the Richards’ equation is a parabolic Eq. (10), but the limit of the semi-continuum model switches between parabolic and hyperbolic type. We are not aware of any research dealing with equations of such type. Since such equations seem to arise naturally by a limiting process of the semi-continuum model, we think they deserve more attention of the soil science and mathematical community.

## Supplementary Information

Supplementary Information.

## Data Availability

No experimental data were generated or analysed during the current study. The code that produced the simulations is available in MatLab upon request from the corresponding author.

## References

[1] Lake L (1989). Enhanced Oil Recovery.

[2] DiCarlo DA (2013). Stability of gravity-driven multiphase flow in porous media: 40 years of advancements. Water Resour. Res..

[3] Xiong Y (2014). Flow of water in porous media with saturation overshoot: A review. J. Hydrol..

[4] Kmec J, Fürst T, Vodák R, Šír M (2019). A semi-continuum model of saturation overshoot in one dimensional unsaturated porous media flow. Sci. Rep..

[5] Dullien FA (1992). L.2 Capillarity in Porous Media.

[6] Or D (2008). Scaling of capillary, gravity and viscous forces affecting flow morphology in unsaturated porous media. Adv. Water Resour..

[7] Miller EE, Miller RD (1956). Physical theory for capillary flow phenomena. J. Appl. Phys..

[8] Parker JC, Lenhard RJ (1987). A model for hysteretic constitutive relations governing multiphase flow: 1. Saturation-pressure relations. Water Resour. Res..

[9] Nimmo JR, Landa ER (2005). The soil physics contributions of edgar buckingham. Soil Sci. Am. J..

[10] Konangi S, Palakurthi NK, Karadimitriou NK, Comer K (2021). Comparison of pore-scale capillary pressure to macroscale capillary pressure using direct numerical simulations of drainage under dynamic and quasi-static conditions. Adv. Water Resour..

[11] Larson RG, Morrow NR (1981). Effects of sample size on capillary pressures in porous media. Powder Technol..

[12] Mishra BK, Sharma MM (1988). Measurement of pore size distributions from capillary pressure curves. Am. Inst. Chem. Eng. J..

[13] Hunt AG, Ewing RP, Horton R (2013). What’s wrong with soil physics. Soil Sci. Soc. Am. J..

[14] Silva MLN, Libardi PL, Gimenes FHS (2018). Soil water retention curve as affected by sample height. Rev. Bras. Cienc. Solo.

[15] Esmaeilpour M, Ghanbarian B, Liang F, Liu H-H (2021). Scale-dependent permeability and formation factor in porous media: Applications of percolation theory. Fuel.

[16] Bear J (1972). Dynamics of Fluids in Porous Media.

[17] Hunt AG, Sahimi M (2017). Flow, transport, and reaction in porous media: Percolation scaling, critical-path analysis, and effective medium approximation. Rev. Geophys..

[18] Glass RJ, Yarrington L (1989). Analysis of wetting front instability using modified invasion percolation theory. Eos Trans. AGU.

[19] Buckingham E (1907). Studies on the Movement of Soil Moisture Bulletin 38.

[20] Lenormand R, Zarcone C, Sarr A (1983). Mechanisms of the displacement of one fluid by another in a network of capillary ducts. J. Fluid Mech..

[21] Wilkinson D (1986). Percolation effects in immiscible displacement. Phys. Rev. A.

[22] Blunt MJ, Scher H (1995). Pore-level modelling of wetting. Phys. Rev. E.

[23] Germann PF (2021). Hess opinions: Unsaturated infiltration—the need for a reconsideration of historical misconceptions. Hydrol. Earth Syst. Sci..

[24] Luckner L, van Genuchten MT, Nielsen DR (1989). A consistent set of parametric models for the two-phase flow of immiscible fluids in the subsurface. Water Resour. Res..

[25] Šimůnek J, Jarvis NJ, van Genuchten M, Gärdenäs A (2003). Review and comparison of models for describing non-equilibrium and preferential flow and transport in the vadose zone. J. Hydrol..

[26] Jarvis NJ (2007). A review of non-equilibrium water flow and solute transport in soil macropores: Principles, controlling factors and consequences for water quality. Eur. J. Soil Sci..

[27] Köhne JM, Köhne S, Šimůnek J (2009). A review of model applications for structured soils: (A) water flow and tracer transport. J. Contamin. Hydrol..

[28] Liu H-H, Zhang R, Bodvarsson GS (2005). An active region model for capturing fractal flow patterns in unsaturated soils: Model development. J. Contamin. Hydrol..

[29] Liu H-H (2017). Fluid Flow in the Subsurface: History, Generalization and Applications of Physical Laws.

[30] Furnari L (2021). Asynchronous cellular automata subsurface flow simulations in two- and three-dimensional heterogeneous soils. Adv. Water Resour..

[31] Richards LA (1931). Capillary conduction of liquid through porous media. Physics.

[32] Bauters TWJ, DiCarlo DA, Steenhuis T, Parlange J-Y (2000). Soil water content dependent wetting front characteristics in sands. J. Hydrol..

[33] Baver CE (2014). Capillary pressure overshoot for unstable wetting fronts is explained by Hoffman’s velocity-dependent contact-angle relationship. Water Resour. Res..

[34] Hassanizadeh SM, Gray WG (1990). Mechanics and thermodynamics of multiphase flow in porous media including interphase boundaries. Adv. Water Resour..

[35] Hassanizadeh SM, Celia MA, Dahle HK (2002). Dynamic effects in the capillary pressure-saturation relationship and its impact on unsaturated flow. Vadose Zone J..

[36] Eliassi M, Glass RJ (2001). On the continuum-scale modeling of gravity-driven fingers in unsaturated porous media: The inadequacy of the Richards equation with standard monotonic constitutive relations and hysteretic equations of state. Water Resour. Res..

[37] Eliassi M, Glass RJ (2002). On the porous-continuum modeling of gravity-driven fingers in unsaturated materials: Extension of standard theory with a hold-back-pile-up effect. Water Resour. Res..

[38] Eliassi M, Glass RJ (2003). On the porous continuum-scale modeling of gravity-driven fingers in unsaturated materials: Numerical solution of a hypodiffusive governing equation that incorporates a hold-back-pile-up effect. Water Resour. Res..

[39] Cueto-Felgueroso L, Juanes R (2009). A phase field model of unsaturated flow. Water Resour. Res..

[40] Gomez H, Cueto-Felgueroso L, Juanes R (2013). Three-dimensional simulation of unstable gravity-driven infiltration of water into a porous medium. J. Comput. Phys.

[41] Schneider M, Köppl T, Helmig R, Steinle R, Hilfer R (2017). Stable propagation of saturation overshoots for two-phase flow in porous media. Transp. Porous Media.

[42] Zhang H, Zegeling PA (2017). A numerical study of two-phase flow models with dynamic capillary pressure and hysteresis. Transp. Porous Media.

[43] Brindt N, Wallach R (2017). The moving-boundary approach for modeling gravity-driven stable and unstable flow in soil. Water Resour. Res..

[44] Brindt N, Wallach R (2020). The moving-boundary approach for modeling 2D gravity-driven stable and unstable flow in partially wettable soils. Water Resour. Res..

[45] DiCarlo DA, Aminzadeh B, Dehghanpour H (2011). Semicontinuum model of saturation overshoot and gravity-driven fingering in porous media. Water Resour. Res..

[46] Kmec J, Fürst T, Vodák R, Šír M (2021). A two dimensional semi-continuum model to explain wetting front instability in porous media. Sci. Rep..

[47] DiCarlo DA (2004). Experimental measurements of saturation overshoot on infiltration. Water Resour. Res..

[48] DiCarlo DA (2007). Capillary pressure overshoot as a function of imbibition flux and initial water content. Water Resour. Res..

[49] Young T (1805). An essay on the cohesion of fluids. Philos. Trans. R. Soc. Lond..

[50] Van Genuchten MT (1980). A closed-form equation for predicting the hydraulic conductivity of unsaturated soils. Soil Sci. Soc. Am. J..

[51] Gawin D, Lefik M, Schrefler BA (2001). ANN approach to sorption hysteresis within a coupled hygro-thermo-mechanical FE analysis. Int. J. Numer. Method Eng..

[52] Beliaev AY, Hassanizadeh SM (2001). A theoretical model of hysteresis and dynamic effects in the capillary relation for two-phase flow in porous media. Transp. Porous Media.

[53] Abreu E, Bustos A, Ferraz P, Lambert W (2019). A relaxation projection analytical-numerical approach in hysteretic two-phase flows in porous media. J. Sci. Comp..

[54] Mualem Y (1974). A conceptual model of hysteresis. Water Resour. Res..

[55] Lenhard RJ, Parker JC (1987). A model for hysteretic constitutive relations governing multiphase flow: 2. permeability-saturation relations. Water Resour. Res..

[56] Visintin A (1994). Differential Models of Hysteresis.

[57] Schweizer B (2017). Hysteresis in porous media: Modelling and analysis. Interfaces Free Bound..

[58] Jang J, Narsilio GA, Santamarina JC (2011). Hydraulic conductivity in spatially varying media: A pore-scale investigation. Geophys. J. Int..

[59] DiCarlo DA (2010). Can continuum extensions to multiphase flow models describe preferential flow?. Vadose Zone J..

[60] Glass RJ, Parlange J-Y, Steenhuis TS (1988). Wetting front instability as a rapid and farreaching hydrologic process in the vadose zone, rapid and farreaching hydrologic processes in the vadose zone. J. Contamin. Hydrol..

[61] Glass RJ, Parlange J-Y, Steenhuis TS (1989). Mechanism for finger persistence in homogenous unsaturated, porous media: Theory and verification. Soil Sci..

[62] Glass RJ, Parlange J-Y, Steenhuis TS (1989). Wetting front instability. 1. Theoretical discussion and dimensional analysis. Water Resour. Res..

[63] Glass RJ, Oosting GH, Steenhuis TS (1989). Preferential solute transport in layered homogeneous sands as a consequence of wetting front instability. J. Hydrol..

[64] Rezanezhad F, Vogel H-J, Roth K (2006). Experimental study of fingered flow through initially dry sand. Hydrol. Earth Syst. Sci. Discuss..

[65] Pales AR (2018). Preferential flow systems amended with biogeochemical components: Imaging of a two-dimensional study. Hydrol. Earth Syst. Sci..

[66] Fatt I (1956). The network model of porous media: I. Capillary pressure characteristics. Pet. Trans. Am. Inst. Min. Metall. Eng..

[67] Zhou D, Stenby EH (1993). Interpretation of capillary-pressure curves using invasion percolation theory. Transp. Porous Media.

[68] Perfect E (2004). Capillary pressure-saturation relations for saprolite: Scaling with and without correction for column height. Vadose Zone J..

[69] Ghanbarian B, Taslimitehrani V, Dong G, Pachepsky YA (2015). Sample dimensions effect on prediction of soil water retention curve and saturated hydraulic conductivity. J. Hydrol..

[70] Pražák J, Šír M, Tesař M (1999). Retention cruve of simple capillary networks. J. Hydrol. Hydromech..

[71] Wang M, Kong L, Zang M (2015). Effects of sample dimensions and shapes on measuring soil-water characteristic curves using pressure plate. J. Rock Mech. Geotech. Eng..

[72] Osher S, Hafez M, Whitlow W (1985). Entropy condition satisfying approximations for the full potential equation of transonic flow. Math. Comput..

[73] Krasnosel’skii MA, Pokrovskii AV (1983). Systems with Hysteresis (Russian).

[74] Mayergoyz ID (1991). Mathematical Models for Hysteresis.

[75] Krejčí P (1996). Hysteresis, Convexity and Dissipation in Hyperbolic Equations.

[76] Kouznetsova V, Brekelmans WAM, Baaijens FPT (2001). An approach to micro-macro modeling of heterogeneous materials. Comput. Mech..

[77] White JA, Borja RI, Fredrich JT (2006). Calculating the effective permeability of sandstone with multiscale lattice Boltzmann/finite element simulations. Acta Geotech..

[78] Al-Raoush R, Papadopoulos A (2010). Representative elementary volume analysis of porous media using X-ray computed tomography. Powder Technol..

[79] Al-Raoush R (2012). Change in microstructure parameters of porous media over representative elementary volume for porosity. Particul. Sci. Technol..

[80] O’Connel ST, Thompson PA (1995). Molecular dynamics-continuum hybrid computations: A tool for studying complex fluid flows. Phys. Rev. E.

[81] Battiato I, Tartakovsky DM, Tartakovsky AM, Scheibe TD (2011). Hybrid models of reactive transport in porous and fractured media. Adv. Water Resour..

[82] Tang Y, Valocchi AJ, Werth CJ (2015). A hybrid pore-scale and continuum-scale model for solute diffusion, reaction, and biofilm development in porous media. Water Resour. Res..

[83] Hesse F, Radu FA, Thullner M, Attinger S (2009). Upscaling of the advection-diffusion-reaction equation with monod reaction. Adv. Water Resour..

[84] Battiato I, Tartakovsky DM (2011). Applicability regimes for macroscopic models of reactive transport in porous media. J. Contamin. Hydrol..

[85] Park HS, Liu WK (2004). An introduction and tutorial on multiple-scale analysis in solids. Comput. Methods Appl. Mech. Eng..

[86] Botan A, Ulm RJ-MP, Coasne B (2015). Bottom-up model of adsorption and transport in multiscale porous media. Phys. Rev. E.

[87] Tartakovsky AM, Tartakovsky DM, Scheibe TD, Meakin P (2008). Hybrid simulations of reaction-diffusion systems in porous media. J. Sci. Comput..

[88] Chu J, Engquist B, Prodanovic M, Tsai R (2011). A Multiscale Method Coupling Network and Continuum Models in Porous Media I Single Phase Flow.

