# Supplementary material for: The difference between semi-continuum model and Richards’ equation for unsaturated porous media flow
Source: Sci Rep. 2022 May 10;12:7650. doi: 10.1038/s41598-022-11437-9 (PMC9090790; doi:10.1038/s41598-022-11437-9)
Supplement: Supplementary file 1 — Supplementary Information. [file 41598_2022_11437_MOESM1_ESM.pdf]

The difference between semi-continuum model and Richards' equation for unsaturated porous media flow

Rostislav Vodák[1], Tomáš Fürst[1], Miloslav Šír[2], and Jakub Kmec[2,*]

[1]Palacký University in Olomouc, Dpt. Mathematical Analysis and Applications of Mathematics, Faculty of Science, Olomouc, 779 00, Czech Republic
[2]Palacký University in Olomouc, Joint Laboratory of Optics, Faculty of Science, Olomouc, 772 07, Czech Republic
[*]jakub.kmec@upol.cz

Limit derivation of the semi-continuum model

With the scaling of the retention curve mentioned in the main article, we can introduce the limit of the semi-continuum model. The limit of the model derived here is a formal one, i.e. we *assume* the solution of the semi-continuum model converges as $\Delta x \to 0$ to a function and show which equation this function should satisfy.

The semi-continuum model consists of two equations (1), (2):

$$\frac{\theta}{\Delta t}\left[S_i(t) - S_i(t-\Delta t)\right] = \frac{1}{\Delta x}\left[q_{i-1,i}(t-\Delta t) - q_{i,i+1}(t-\Delta t)\right], \tag{1}$$

$$q_{i,i+1}(t) = \frac{\kappa}{\mu}\sqrt{k(S_i(t))k(S_{i+1}(t))}\left(\rho g - \frac{P_{i+1}(t) - P_i(t)}{\Delta x}\right). \tag{2}$$

Equation (1) is an explicit discretized version of equation (3):

$$\theta \partial_t S_i(t) = \frac{1}{\Delta x}\left[q_{i-1,i}(t) - q_{i,i+1}(t)\right]. \tag{3}$$

In these equations, S [-] is saturation, P [Pa] is capillary pressure and $q_{i,i+1}$ [m/s] denotes the flux between the blocks i and $i+1$. θ [-] denotes the porosity of the material, κ [m^2] the intrinsic permeability, ρ [kg/m^3] the fluid density, g [m/s^2] acceleration due to gravity, and μ [Pas] the dynamic viscosity of fluid. Two material characteristics are used in the semi-continuum model, the retention curve and the relative permeability function $k(S)$ $[-]$. In the case of a retention curve, hysteresis is considered.

The main branches of the retention curve of a block take the form

$$P_w(h,S) = h\left(-100\log\left(\frac{1}{S} - 1\right)\right) + C_1, \tag{4}$$

for the main wetting branch, and

$$P_d(h,S) = h\left(-100\log\left(\frac{1}{S} - 1\right)\right) + C_2, \tag{5}$$

for the main draining branch, where C_1 [Pa] and C_2 [Pa] are constants. The parameter h [m] is the scaling parameter equals to the block size Δx.

Let us now complete the model (equations (1), (2)) by stating the initial conditions

$$S_i(0) = S_{i,in} \quad \text{for} \quad i = 0, \dots, N, \tag{6}$$

and boundary conditions

$$q_{-1,0}(t) = q_0 > 0 \quad \text{and} \quad q_{N,N+1}(t) = 0. \tag{7}$$

The zero-flux boundary condition at the bottom boundary is not necessary and can be replaced by another one without a significant effect on the limit equation. Let us denote by x_{i+1} the point on the boundary between blocks i and $i+1$. Points x_0 and x_{N+1} form the boundary of the one-dimensional sample. We put $h = \Delta x$. The $i-$th block thus corresponds to the interval $[x_i, x_i + h)$. Furthermore, we define the following notation. A function $\tilde{f}_h(x)$ denotes a piecewise constant function that takes the value f_i on each interval $x \in [x_i, x_i + h)$. In this notation, $\tilde{S}_h(x,t)$ is the saturation, piecewise constant on each block. A piecewise linear (and thus continuous on $[0,L]$) approximation will be denoted without the tilde, i.e.

$$f_h(x) = \left(1 + \frac{x_i}{h} - \frac{x}{h}\right) f_i + \left(\frac{x}{h} - \frac{x_i}{h}\right) f_{i+1},$$

for $x \in [x_i, x_i + h)$. In the following paragraphs, we will use these piecewise constant and piecewise linear approximations to saturation, pressure, and flux. Equation (3) can be rewritten as a partial differential equation

$$\theta \partial_t \tilde{S}_h(x,t) = -\partial_x q_h(x,t), \qquad \text{for } (x,t) \in [0,L] \times [0,T], \tag{8}$$

where we used

$$f_i = q_{i-1,i} \quad \text{and} \quad f_{i+1} = q_{i,i+1}.$$

This approach is inspired by Rothe's scheme which relates a partial differential equation to its discrete approximation[1]. Similarly, equation (2) can be rewritten as follows:

$$\tilde{q}_h(x+h,t) = \frac{\kappa}{\mu} \sqrt{k(\tilde{S}_h(x,t))} \sqrt{k(\tilde{S}_h(x+h,t))} \left(\rho g - \partial_x P_h(h,x,t)\right) \qquad \text{for } (x,t) \in [0,L] \times [0,T], \tag{9}$$

where P_h is the hysteresis operator generated by equations (4) and (5).

Next, we employ the toolbox of modern partial differential equation theory and pass to the weak formulation. We take a smooth function φ with compact support, multiply both sides of equation (8) by it, and integrate over the interval $[0,L]$ to get

$$\theta \int_0^L \partial_t \tilde{S}_h(x,t) \varphi(x)\, dx = \int_0^L q_h(x,t) \partial_x \varphi(x)\, dx + q_0 \varphi(0). \tag{10}$$

In this equation, the derivative is transferred to the test function φ which enables us to relax the assumptions on the limits performed below.

The retention curve defined by equations (4) and (5) can be decomposed into two branches (wetting and draining) in the following symmetrical way:

$$P_w(h,S) = f(h,S) + C, \qquad \text{and} \qquad P_d(h,S) = f(h,S) - C, \tag{11}$$

with

$$f(h,S) = h\left(-100 \log\left(\frac{1}{S} - 1\right)\right) + \frac{C_1 + C_2}{2}$$

and $C = \frac{C_1 - C_2}{2}$. The scanning curves are modelled as line segments with the derivative K_{PS}.

Let us note that the constant K_{PS} is fixed during the limiting process. Using the form of the two branches, we can see the similarity between equation (11) and the classical Prandtl model of elasto-plasticity (the stop operator), see equations $(2.2)-(2.5)$ (p. $15-16$) in Visintin for more details[2]. The hysteresis operator $P(h)$ defined by equation (11) and the scanning curves can be thus expressed using the differential inequality

$$\left(K_{PS} \partial_t S - \partial_t P(h)\right)\left(P(h) - v\right) \geq 0, \qquad \text{for all } v \in [P_d(h,S), P_w(h,S)],$$

$$\text{with } \; P(h) \in [P_d(h,S), P_w(h,S)], \qquad \text{and} \qquad S \in [0,1].$$

To see the similarity with the classical Prandtl model of elasto-plasticity, we use the substitution

$$U(h,S) := K_{PS} S - f(h,S), \; V(h) := P(h) - f(h,S), \; w := v - f(h,S). \tag{12}$$

The resulting operator form of the semi-continuum model takes the form of the following differential inequality

$$\left(\partial_t U(h,S) - \partial_t V(h)\right)\left(V(h) - w\right) \geq 0, \qquad \text{for all } w \in [-C,C] \qquad \text{with } V(h) \in [-C,C]. \tag{13}$$

We now check whether the differential inequality in equation (13) corresponds to the hysteresis operator defined by equation (11) with the scanning curves:

1. Let $V(h) \in (-C,C)$. Then there exist $w_j \in [-C,C]$, $j=1,2$, such that $V(h)-w_1>0$ and $V(h)-w_2<0$. From equation (13) it follows:
$$\partial_t U(h,S) - \partial_t V(h) = 0.$$
Integration in time variable together with equation (12) results in
$$P(h,t) = K_{PS}S(t) - K_{PS}S(t_0) + P(h,t_0),$$
where t_0 is an initial time such that $P(h,t_0) \in [P_d(h,S(t_0)), P_w(h,S(t_0))]$. Thus, we are located on the scanning curve for this case.

2. Let $V(h)=C$ and thus $P(h)=f(h,S)+C$. Then $V(h)-w \geq 0$ and $\partial_t U(h,S) - \partial_t V(h) \geq 0$. Hence and from equation (12) it follows
$$K_{PS}\partial_t S \geq \partial_t P(h) \Rightarrow K_{PS} \geq \partial_S f(h,S),$$
because the pressure corresponds to this branch only if $\partial_t S \geq 0$ and moreover $\partial_t f(h,S) = \partial_S f(h,S)\partial_t S$. Without the inequality $\partial_t S \geq 0$ we are unable to connect the scanning curves with the wetting branch. From the inequality $K_{PS} \geq \partial_S f(h,S)$ it follows that the scanning curves and the wetting branch are connected.

3. Let $V(h) = -C$ and thus $P(h) = f(h,S) - C$. Then $V(h)-w \leq 0$ and $\partial_t U(h,S) - \partial_t V(h) \leq 0$. Hence and from equation (12) it follows
$$K_{PS}\partial_t S \leq \partial_t P(h) \Rightarrow K_{PS} \geq \partial_S f(h,S),$$
because the pressure corresponds to this branch only if $\partial_t S \leq 0$ and moreover $\partial_t f(h,S) = \partial_S f(h,S)\partial_t S$. Without the inequality $\partial_t S \leq 0$ we are unable to connect the scanning curves with the draining branch. From the inequality $K_{PS} \geq \partial_S f(h,S)$ it follows that the scanning curves and the draining branch are connected.

Finally, assume that as $h \to 0$, the solutions of the model derived above and the corresponding operator converge in the following sense
$$\begin{aligned}&\tilde{S}_h \to S, \quad \partial_t \tilde{S}_h \to \partial_t S, \quad q_h \to q, \quad \tilde{q}_h \to q, \quad V(h) \to P_H - \frac{C_1+C_2}{2},\\ &\partial_x P_h(h) \to \partial_x P_H, \quad \partial_t P_h(h) \to \partial_t P_H \quad \text{a.e. in } [0,L]\times[0,T].\end{aligned} \tag{14}$$
The validity of this assumption is suggested by the numerical evidence described in the main article. Assume further we can apply Lebesgue's dominated convergence theorem to equation (10). Performing the limit in equations (10) and (11) yields
$$\theta \int_0^L \partial_t S(x,t)\varphi(x)\,dx = \int_0^L q(x,t)\partial_x \varphi(x)\,dx + q_0\varphi(0), \tag{15}$$
where
$$q := \frac{\kappa}{\mu}\sqrt{k(S^-)}\sqrt{k(S^+)}\left(\rho g - \partial_x P_H\right), \qquad S^{\pm}(x_0,t) = \lim_{x\to x_0^{\pm}} S(x,t) \tag{16}$$
and
$$\left(K_{PS}\partial_t S - \partial_t P_H\right)\left(P_H - v\right) \geq 0, \qquad \text{for all } v \in [C_2,C_1], \qquad \text{and } P_H \in [C_2,C_1]. \tag{17}$$
Thus, the limit form of the semi-continuum model is a weak formulation (15) for a partial differential equation together with the classical Buckingham-Darcy law (16), containing a hysteresis operator of the Prandtl model of elasto-plasticity (17). Passing from the weak formulation to the classical one yields
$$\theta \partial_t S + \partial_x\left(\frac{\kappa}{\mu}\sqrt{k(S^-)}\sqrt{k(S^+)}(\rho g - \partial_x P_H)\right) = 0. \tag{18}$$
Equations (17)–(18) represent the classical form of the *limit of the semi-continuum model.* It is a partial differential equation with a hysteresis operator of Prandtl-type under the derivative. If we are located on the main wetting or draining branches, the limit will be a hyperbolic differential equation, because the pressure saturation relation is constant and thus independent on the space variable. This makes the limit switch between parabolic and hyperbolic type. Let us note that in the case of continuous saturation, it applies $k(S) = \sqrt{k(S^-)}\sqrt{k(S^+)}$.

References

1. Roubíček, T. *Nonlinear Partial Differential Equations with Applications* (Birkhäuser Basel, 2013).

2. Visintin, A. *Differential Models of Hysteresis.* (New York: Springer., 1994).
